# Target of rapamycin signaling regulates high mobility group protein association to chromatin, which functions to suppress necrotic cell death

**DOI:** 10.1186/1756-8935-6-29

**Published:** 2013-09-02

**Authors:** Hongfeng Chen, Jason J Workman, Alexa Tenga, R Nicholas Laribee

**Affiliations:** 1 Department of Pathology and Laboratory Medicine and Center for Cancer Research, University of Tennessee Health Science Center, Memphis, TN 38163, USA

**Keywords:** High mobility group protein, Histone, Necrosis, Nutrient signaling, Target of rapamycin

## Abstract

**Background:**

The target of rapamycin complex 1 (TORC1) is an evolutionarily conserved signal transduction pathway activated by environmental nutrients that regulates gene transcription to control cell growth and proliferation. How TORC1 modulates chromatin structure to control gene expression, however, is largely unknown. Because TORC1 is a major transducer of environmental information, defining this process has critical implications for both understanding environmental effects on epigenetic processes and the role of aberrant TORC1 signaling in many diseases, including cancer, diabetes, and cardiovascular disease.

**Results:**

To elucidate the role of TORC1 signaling in chromatin regulation, we screened a budding yeast histone H3 and H4 mutant library using the selective TORC1 inhibitor rapamycin to identify histone residues functionally connected to TORC1. Intriguingly, we identified histone H3 lysine 37 (H3K37) as a residue that is essential during periods of limited TORC1 activity. An H3K37A mutation resulted in cell death by necrosis when TORC1 signaling was simultaneously impaired. The induction of necrosis was linked to alterations in high mobility group (HMG) protein binding to chromatin. Furthermore, the necrotic phenotype could be recapitulated in wild-type cells by deregulating the model HMG proteins, Hmo1 or Ixr1, thus implicating a direct role for HMG protein deregulation as a stimulus for inducing necrosis.

**Conclusions:**

This study identifies histone H3 and H4 residues functionally required for TORC1-dependent cell growth and proliferation that are also candidate epigenetic pathways regulated by TORC1 signaling. It also demonstrates a novel role for H3K37 and TORC1 in regulating the binding of select HMG proteins to chromatin and that HMG protein deregulation can initiate a necrotic cell death response. Overall, the results from this study suggest a possible model by which chromatin anchors HMG proteins during periods of limited TORC1 signaling, such as that which occurs during conditions of nutrient stress, to suppress necrotic cell death.

## Background

Environmental influences on eukaryotic cell growth, proliferation, and development have well-documented effects on epigenetic processes [1,2]. However, the mechanisms underlying the regulation of epigenetic, or chromatin, pathways by the environment remains largely unknown [1,2]. Chromatin structure and function is modulated by factors controlling the packaging of DNA with histone proteins, including histone post-translational modifications, DNA methylation, ATP-dependent chromatin-remodeling enzymes, noncoding RNA, and the incorporation of histone variants [3]. Although considerable progress defining the biochemical pathways regulating chromatin has been made, determining how these pathways are themselves controlled by environmental stimuli remains a major challenge in the field of epigenetics.

The eukaryotic target of rapamycin (TOR) pathway is an environmentally regulated signaling pathway activated by nutrients (predominantly amino acids), growth factors, and energy states to promote anabolic processes necessary for cell growth and proliferation while actively suppressing such catabolic processes as autophagy [4,5]. While TOR signals through two effector branches, the TOR complex 1 (TORC1) and TOR complex 2 (TORC2) pathways [6,7], only TORC1 is directly regulated by nutrient signals. TORC1 signaling regulates the transcriptional and translational apparatuses that promote growth and proliferation and this complex is inhibited by the macrolide rapamycin. Genetic analyses in yeast have identified many TORC1 pathway members since mutations in genes coding for these factors typically modify sensitivity to rapamycin [5,8]. In yeast, TORC1 consists of either the Tor1 or Tor2 kinases, Kog1 (a homolog to mammalian Raptor), Lst8 and Tco89 subunits [5]. Intravacuolar amino acid levels are registered by the EGO complex, which consists of the Ego1 and Ego3 proteins along with the Rag GTPases Gtr1 and Gtr2. EGO then recruits TORC1 to the vacuole membrane, resulting in TORC1 activation. TORC1 then signals to downstream effectors to regulate transcriptional and translational processes controlling cell growth and proliferation [9]. The best-characterized TORC1 target in yeast is the AGC kinase family member Sch9, which is a functional ortholog to the Akt/PKB and ribosomal S6 kinases [10]. Sch9 is phosphorylated on multiple serine and threonine residues by TORC1, resulting in Sch9 activation [11]. Sch9 then phosphorylates a number of downstream factors involved in diverse biological processes, including regulators of ribosomal transcription, mRNA export, and protein translation [12].

The Tor1 kinase can also be recruited to the promoter regions of the RNA polymerase I and III (Pol I and Pol III) transcribed 35S and 5S ribosomal DNA (rDNA) genes, respectively, to activate their transcription [13,14]. This process is also conserved in mammals, as mTor directly regulates transcription by all three RNA polymerases [15-17]. Transcriptional regulation requires significant alterations to chromatin structure and function but how TORC1 regulates chromatin to mediate its transcriptional effects has yet to be defined in detail. Previous studies have linked TORC1 to both the RSC chromatin-remodeling complex [18] and the Esa1 histone acetyltransferase [19]. In addition, TORC1 activation of Sch9 leads to phosphorylation of the transcriptional repressors Stb3, Dot6, and Tod6 and the disruption of Rpd3-dependent histone deacetylase repression of ribosomal protein (RP) and ribosomal biogenesis (Ribi) genes [20]. TORC1 has also been functionally linked to Rpd3, independently of these transcriptional repressors [21]. However, the full extent of TORC1 signaling to the epigenome, and the underlying mechanisms involved, remains to be determined.

We have pursued a chemical genomics approach to probe how TORC1 interacts with chromatin as an initiation point for identifying mechanisms by which the environment influences the epigenome. Utilizing a library of histone H3 and H4 mutants [22], we have identified a number of individual histone residues, many of which are known to be post-translationally modified, that are functionally linked to TORC1 signaling. We have characterized in detail a subset of these mutants and identified an essential role for histone H3 lysine 37 (H3K37) in anchoring select high mobility group (HMG) proteins to chromatin during periods of limited TORC1 activity. Disruption of this histone-HMG contact residue combined with reduced TORC1 signaling resulted in cell death by necrosis. Furthermore, necrosis was recapitulated in an otherwise wild-type cell by the transient, individual overexpression of multiple HMG proteins, suggesting a direct role for HMG protein deregulation in inducing necrosis.

## Results

### Identification of histone H3 and H4 residues functionally linked to TORC1

To identify histone H3 and H4 residues genetically connected to TORC1 signaling, we screened a histone H3/H4 mutant library using a sub-inhibitory (25 nM) concentration of rapamycin to identify those mutants exhibiting altered rapamycin sensitivity. Each mutant was screened at least twice and the results were scored as described in the Methods. For each mutant exhibiting a reproducible rapamycin phenotype, we also analyzed any other amino acid substitutions at that position that were available in the library. For example, mutations at lysine residues prompted us to analyze corresponding lysine to arginine (which restores positive charge but prevents acetylation) and lysine to glutamine (which mimics constitutive acetylation) changes. The results for histone H3 and histone H4 are summarized in Tables 1 and 2, along with their positions in the nucleosome as annotated in the HistoneHits database [23]. Intriguingly, 30 (≈26%) H3 and 13 (≈14%) H4 mutants demonstrated altered rapamycin phenotypes, most of which were sensitive (≈73% of all hits for histone H3 and ≈92% of all hits for histone H4, see Tables 1 and 2). A much smaller percentage of resistant mutants (≈27% for histone H3 and ≈8% for histone H4) were identified. In general, more robust rapamycin phenotypes, either sensitive or resistant, were identified for histone H3 mutants relative to histone H4, which is also consistent with the overall screening results. These data suggest histone H3 may have a greater functional role in TORC1 signaling relative to histone H4.

**Table 1:** Histone H3 mutants exhibiting altered rapamycin sensitivity

**Histone H3 residue**	**Rapamycin phenotype**	**Nucleosome location**	**Source**
A1S	−1	Tail	This study
T3A	+1	Tail	This study
T3D	0	Tail	This study
Q5A	+2	Tail	This study
Q5E	+2	Tail	This study
K14A	−2	Tail	[36]
K14R	0	Tail	This study
K14Q	0	Tail	This study
R17A	−2	Tail	This study
R17K	−2	Tail	This study
K18A	−1	Tail	[36]
K18R	−2	Tail	This study
K18Q	0	Tail	This study
K23A	−1	Tail	[36]
K23R	0	Tail	This study
K23Q	0	Tail	This study
S28A	−2	Tail	This study
S28D	0	Tail	This study
P30A	−1	Tail	This study
K37A	Lethal	Tail	This study
K37R	0	Tail	This study
K37Q	0	Tail	This study
R49A	−2	Lateral	This study
R49K	0	Lateral	This study
K56A	−3	Lateral	[36]
K56R	−2	Lateral	[36]
K56Q	+2	Lateral	[36]
S57A	+1	Disk	[36]
S57D	+1	Disk	[36]
E73A	−1	Disk	This study
E73Q	−1	Disk	This study
Q76A	−1	Disk	This study
Q76E	−1	Disk	This study
T80A	+1	Disk	This study
T80D	−1	Disk	This study
L82A	−1	Disk	This study
R83A	−2	Lateral	This study
R83K	0	Lateral	This study
Q85A	−2	Lateral	This study
Q85E	0	Lateral	This study
S86A	−2	Lateral	This study
S86D	−2	Lateral	This study
L92A	+1	Buried	This study
E105A	−1	Disk	This study
A110S	−1	Buried	This study
A111S	−1	Buried	This study
A114S	−1	Disk	This study
V117A	−3	Lateral	This study
Q120A	+1	Lateral	This study
K121A	−1	Disk	This study
K121R	0	Disk	This study
K121Q	−1	Disk	This study
K122A	−1	Disk	This study
K122R	−3	Disk	This study
K122Q	−1	Disk	This study
R129A	+1	Disk	This study
R129K	0	Disk	This study
**Total number of H3 mutants screened**			**114**
**Number of H3 mutants with phenotype**			**30**
**Percentage of total H3 mutants with phenotype**			**26.3%**
**Percentage of H3 mutants with resistant phenotypes**			**26.7%**
**Percentage of H3 mutants with sensitive phenotypes**			**73.3%**

**Table 2:** Histone H4 mutants exhibiting altered rapamycin sensitivity

**Histone H4 residue**	**Rapamycin phenotype**	**Nucleosome location**	**Source**
L10A	−1	Tail	This study
G14A	−1	Tail	This study
K20A	−1	Disk	This study
K20R	0	Disk	This study
K20Q	−1	Disk	This study
L22A	−1	Disk	This study
G28A	−1	Disk	This study
I29A	−1	Buried	This study
S47A	−1	Lateral	This study
S47D	+1	Lateral	This study
S64A	−1	Disk	This study
S64D	−1	Disk	This study
I66A	−1	Buried	This study
V81A	+1	Buried	This study
Y88A	−1	Disk	This study
Y88E	0	Disk	This study
Y88F	0	Disk	This study
G94A	−1	Disk	This study
Y98A	−1	Buried	This study
Y98E	−1	Buried	This study
Y98F	0	Buried	This study
**Total number of H4 mutants screened**			**94**
**Number of H4 mutants with phenotype**			**13**
**Percentage of total H4 mutants with phenotype**			**13.8%**
**Percentage of H4 mutants with resistant phenotypes**			**7.7%**
**Percentage of H4 mutants with sensitive phenotypes**			**92.3%**

### Histone H3K14A and H3K37A rapamycin sensitivity exhibit functional differences

**Figure 1:**
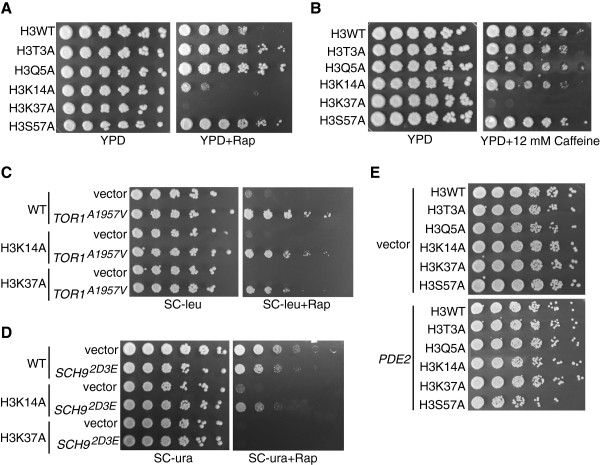
**Selective effects of histone H3 residues on TORC1-regulated cell growth. (A)** Subset of histone H3 residues identified in chemical genomics screen. Equal numbers of cells were 5-fold serially diluted and spotted to control (yeast extract/peptone/dextrose (YPD)) or YPD containing 25 nM rapamycin plates. Plates were incubated at 30°C for 4 days before photographing. **(B)** As **(A),** except cells were spotted to control YPD or YPD plates containing 12 mM caffeine. **(C)** A hyperactive Tor1 kinase (Tor1^A1957V^) rescues H3K14A and H3K37A growth on rapamycin. The indicated strains were transformed with control vector or vector expressing Tor1^A1957V^ and spotted to SC-leucine or SC-leucine containing 25 nM rapamycin. Plates were incubated at 30°C for 4 days. **(D)** Sch9 signaling rescues the H3K14A, but not H3K37A, TORC1 phenotype. The indicated strains were transformed with control vector or vector expressing Sch9^2D3E^, spotted to either SC-uracil or SC-uracil containing 25 nM rapamycin, and incubated as in **(D)**. **(E)** H3S57A mutants are sensitive to decreased protein kinase A (PKA) signaling. Cells carrying control vector or a high copy *PDE2* expression vector were spotted to SC-leucine plates and incubated at 30°C for 4 days. leu, leucine; Rap, rapamycin; SC, synthetic complete medium; ura, uracil; WT, wild-type.

To explore in detail how these mutants affect TORC1-dependent processes, we focused on a subset of histone H3 mutants that resulted in either robust rapamycin resistance (H3T3A, H3Q5A and H3S57A) or sensitivity (H3K14A and H3K37A) relative to the histone H3 wild-type (H3WT) strain. Intriguingly, H3K37A was the only mutant that completely prevented growth on rapamycin (Figure 1A and Table 1), suggesting an essential role for this residue in TORC1 signaling. This rapamycin sensitivity cannot be explained as a loss of H3K36 or H3P38 post-translational modifications, as neither the H3K36A nor the H3P38A mutation were identified in our screen as having significant rapamycin phenotypes (data not shown). We next determined whether these mutations also affected sensitivity to caffeine which is another, albeit less specific, inhibitor of TORC1 [24,25]. Surprisingly, only H3K37A exhibited increased sensitivity to 12 mM caffeine while all other mutants, including H3K14A, grew comparable to the wild-type strain (Figure 1B). To determine whether the H3K14A and H3K37A rapamycin sensitivity could be rescued by increased TORC1 signaling, we transformed the wild-type strain and each mutant with a control vector or a vector expressing a mutant Tor1 kinase (Tor1^A1957V^) that has increased kinase activity *in vitro*[24]. Under these conditions, wild-type cells expressing Tor1^A1957V^ grew considerably better in the presence of rapamycin than wild-type cells carrying the control vector (Figure 1C). This trend was mirrored by the H3K14A mutant, albeit the colony sizes in the Tor1^A1957V^ expressing H3K14A cells were still smaller than wild-type colonies. Interestingly, while expression of Tor1^A1957V^ in the H3K37A mutant did rescue growth, this mutant still grew more poorly than the H3K14A or H3WT (Figure 1C). These differences are unlikely to be caused by differential expression of the *TOR1*^*A1957V*^ plasmid as Tor1^A1957V^ protein levels were equivalent between the strains (Additional file 1). These results demonstrate that the rapamycin sensitivity identified in H3K14A and H3K37A mutants is due specifically to decreased TORC1 activity.

**Figure 2:**
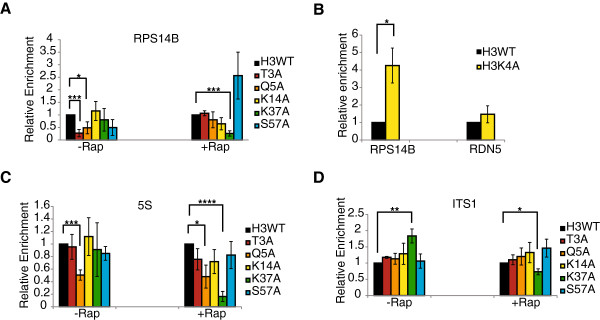
**Histone H3 mutants have differential impacts on TORC1-regulated gene expression.** H3WT and the indicated histone mutants were grown to log phase and then mock treated or treated with 25 nM rapamycin for 1.5 hours. Total RNA was extracted, cDNA synthesized using random hexamer primers, and target gene expression analyzed by qPCR. The expression of all target genes was normalized to the *ACT1* housekeeping gene. **(A)** Expression of the RNA Pol II transcribed ribosomal protein gene, *RPS14B,* in the indicated strains. **(B)** Expression of *RPS14B* and the RNA Pol III transcribed *5S* rRNA were analyzed in H3WT and H3K4A. **(C)***5S* rRNA expression in the indicated histone strains. **(D)** Expression of the RNA Pol I transcribed *ITS1*-containing *35S* rRNA. Individual histone H3 mutants are expressed relative to the H3 wild-type strain (H3WT), which is set to a value of 1. Data are the average and standard deviation of four independent experiments with significance determined by Student’s *t* test. *P < 0.05; **P < 0.01; ***P < 0.005; ****P < 0.0005. Rap, rapamycin.

In budding yeast, TORC1 phosphorylates the downstream Sch9 kinase to mediate some TORC1-regulated functions. To determine whether the rapamycin-induced growth defects in H3K14A and H3K37A mutants were due to decreased Sch9 activation, we transformed wild-type, H3K14A, and H3K37A yeast with control vector or vector expressing the Sch9^2D3E^ mutant. This mutant has acidic amino acid changes at five of the phosphoacceptor sites normally phosphorylated by TORC1, resulting in a Sch9 kinase that is active independently of upstream TORC1 activity [11]. While cells carrying control vector or the Sch9^2D3E^ expression vector grew normally on control plates, we observed markedly different results in the presence of rapamycin. Wild-type cells carrying Sch9^2D3E^ grew more poorly on rapamycin plates when Sch9^2D3E^ was expressed compared with control vector (Figure 1D), although the significance of this growth defect is currently unknown. In the H3K14A mutants, the Sch9^2D3E^ plasmid exhibited a partial reversal of their rapamycin sensitivity, suggesting that the rapamycin-induced growth defects in this mutant are due at least in part to decreased Sch9 activity. Surprisingly, Sch9^2D3E^ did not rescue growth of H3K37A on rapamycin (Figure 1D). This effect was not the result of lower Sch9^2D3E^ expression in the H3K37A mutant relative to wild-type, as Sch9^2D3E^ was expressed comparably in the different strains (Additional file 1).

The Ras/PKA pathway is an essential regulator of cell growth and proliferation that is activated predominantly by glucose availability [26-28]. Ras/PKA and TORC1 also constitute the two main nutrient-regulated signaling pathways in yeast. We next determined whether these histone mutants exhibited sensitivity to reduced Ras/PKA activity, since TORC1 and Ras/PKA signaling have been both positively and negatively linked to each other [26-28]. A control vector or a multicopy vector expressing the high affinity phosphodiesterase *PDE2*, which decreases cAMP levels and reduces PKA signaling [29], was transformed into H3WT and the individual histone mutants. Surprisingly, all histone mutants except for H3S57A tolerated lower PKA signaling comparably to wild-type strains (Figure 1E). The H3S57A mutant exhibited mild sensitivity to reduced PKA activity, suggesting it may be functionally important for the downstream effects of the Ras/PKA pathway. Collectively, these results demonstrate that H3K37A is absolutely essential for growth during conditions of reduced TORC1, but not Ras/PKA, signaling and that this requirement is independent of the activation of the downstream TORC1 effector kinase Sch9.

### TORC1-regulated gene expression is disrupted in a subset of histone H3 mutants

We next asked whether these histone H3 mutants altered TORC1-regulated gene transcription in a manner consistent with their rapamycin phenotype. Since transcription of the RP genes occurs as a regulon [30], for these experiments we analyzed the expression of the RP gene *RPS14B* as a model TORC1-regulated RNA polymerase II (Pol II) transcriptional target. Surprisingly, in the absence of rapamycin, the rapamycin-resistant mutants H3T3A and H3Q5A reduced *RPS14B* expression whereas the other mutants did not have significant effects (Figure 2A). However, upon a 1.5-hour rapamycin treatment, this difference in *RPS14B* expression in H3TA and H3Q5A mutants relative to wild-type strains was erased (Figure 2A). Intriguingly, while the H3K37A mutation did not affect *RPS14B* expression in the absence of rapamycin, there was a dramatic reduction in *RPS14B* expression after rapamycin treatment (Figure 2A). Neither the H3K14A nor the H3S57A mutants significantly affected *RPS14B* expression under either condition (Figure 2A). The decreased *RPS14B* expression in both the H3T3A and H3Q5A mutants is probably not caused by loss of adjacent H3K4 methylation as a H3K4A mutant resulted in increased *RPS14B* expression relative to H3WT (Figure 2B).

We next monitored expression of the RNA Pol I transcribed polycistronic 35S rRNA and the 5S rRNA, which is transcribed by RNA Pol III. The 35S rRNA is co-transcriptionally and post-transcriptionally processed into the mature 18S, 5.8S, and 25S rRNAs, which have relatively long half-lives [31]. Their expression can also be maintained under conditions that significantly reduce the number of rDNA repeats [32]. Since their steady-state levels may not accurately reflect changes to Pol I transcriptional activity, we also analyzed expression of the ITS1 sequence that lies between the 18S and 5.8S rRNA sequences [31]. ITS1 is transiently present in the nascent rRNA but is then rapidly co-transcriptionally cleaved [33,34] and can be utilized as a proxy of Pol I transcription and co-transcriptional 35S processing [35,36]. Expression of the 5S rRNA in the absence of rapamycin was normal for all mutants except H3Q5A, where 5S rRNA levels were significantly reduced (Figure 2C). This reduction was not due to loss of H3K4 methylation, as the H3K4A mutation did not affect 5S expression (Figure 2B). After rapamycin treatment, 5S rRNA levels were still reduced in the H3Q5A mutant; however, under these conditions the H3K37A mutant also exhibited a dramatic reduction in 5S rRNA levels (Figure 2C). Interestingly, ITS1 expression was modestly elevated in the H3K37A mutant approximately two-fold before rapamycin treatment (Figure 2D), suggesting either a modest increase in Pol I transcription or a decrease in co-transcriptional rRNA processing [36]. However, ITS1 levels decreased after rapamycin treatment indicating a reduction in Pol I transcription. Quantification of 18S and 25S rRNA levels revealed only marginal changes in their expression (Additional file 2). These results implicate H3T3 and H3Q5 as having critical roles in the regulation of TORC1-specific gene regulation mediated by Pol II- and Pol III-dependent mechanisms and that H3K37A is critical for TORC1-regulated gene expression when TORC1 activity is reduced.

### H3K37 has a critical role in TORC1-regulated cell-cycle progression

**Figure 3:**
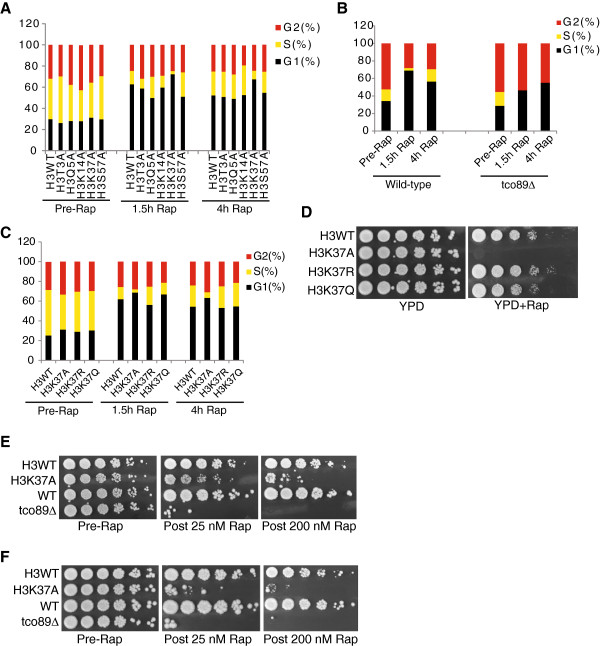
**Histone H3K37A mutants exhibit a rapamycin-induced cell-cycle defect. (A)** H3K37A mutants have defects in S-phase after rapamycin treatment. H3WT and histone mutants were grown asynchronously to log phase before mock treating or treating with 25 nM rapamycin for 1.5 hours and 4 hours. Cells were then stained with SYTOX Green and analyzed by flow cytometry. The percentages of cells in each phase are presented and are listed in Additional file 3. **(B)***tco89Δ* results in a complete absence of S-phase cells after rapamycin treatment. The experiment in (A) was repeated using wild-type (BY4741) and *tco89Δ*. The percentages of cells in each phase are listed in Additional file 4. **(C)** Both H3K37 charge and acetylation mutations rescue the cell-cycle phenotype. Experiment as **(A)**, except H3WT, H3K37A, H3K37R, and H3K37Q mutants were analyzed. Additional file 5 lists the percentages of each cell-cycle phase. **(D)** Strains from (C) were spotted to YPD control plates or YPD plates containing 25 nM rapamycin and incubated at 30°C for four days. **(E,F)** H3K37A and *tco89Δ* mutants exhibit sensitivity to transient rapamycin treatment. The indicated strains were grown to log phase before spotting equal numbers of cells to control YPD plates. The remaining cultures were treated with 25 nM (sub-inhibitory) or 200 nM (inhibitory) rapamycin for 5.5 hours **(E)** or 24 hours **(F)**. Cells were washed, 5-fold serially diluted, spotted to YPD plates, and incubated at 30°C for 3 days. Rap, rapamycin; WT, wild-type.

TORC1 has a well-established role in regulating the G1/S-phase of the cell cycle and a much more poorly understood role in G2/M-phase regulation [5,37,38]. We next determined whether these histone H3 mutants, and in particular H3K37A, affected the cell cycle in a TORC1-dependent manner. Asynchronous cultures of wild-type yeast and the individual histone mutants were grown to log phase, mock treated or treated with 25 nM rapamycin for 1.5 hours and 4 hours before staining with SYTOX Green and analysis by flow cytometry. No significant differences in the cell-cycle profiles of wild-type cells and histone mutants were detected in the mock-treated populations (Figure 3A, Additional file 3). However, a 1.5-hour rapamycin treatment resulted in an enrichment of the wild-type strain and mutants in G1 relative to S- and G2/M phases but with significant heterogeneity in their distribution. Wild-type, H3T3A, and H3K14A had similar G1-phase distributions (approximately 63%, 59%, and 60% respectively; see Additional file 3). However, the H3Q5A and H3S57A mutations resulted in only approximately 50% of the cells in G1 (Figure 3A and Additional file 3). While only minor differences were detected in the G2/M populations, significant differences were observed in S-phase. Specifically, the wild-type strain H3T3A and H3K14A mutants were distributed similarly (approximately 13%, 9%, and 11% respectively) while the H3Q5A and H3S57A mutants had increased S-phase populations (approximately 20% and 23% respectively). These differences were largely erased by 4 hours of rapamycin treatment, however (Figure 3A, Additional file 3). While H3K37A had a similar distribution of cells in G2/M relative to wild-type at 1.5 and 4 hours post-rapamycin treatment, there were significantly more cells in G1 (approximately 72%) and far fewer cells in S-phase (3%) suggesting that H3K37A was not transitioning from G1 into S-phase efficiently (Figure 3A and Additional file 3).

We next asked whether a TORC1 mutant (*tco89Δ*) that causes rapamycin hypersensitivity also affected cell-cycle regulation comparable to the H3K37A mutation. The experiment described in Figure 3A was repeated using wild-type and *tco89Δ* cells. While the pre-rapamycin wild-type and *tco89Δ* populations were similarly distributed throughout the cell cycle, the 1.5 and 4-hour post-rapamycin samples exhibited dramatic differences (Figure 3B). The S-phase population in the wild-type strain was reduced significantly at 1.5 hours (approximately 3%) but increased to 14% by 4 hours whereas the *tco89Δ* mutant had no detectable cells in S-phase at either 1.5 or 4 hours (Figure 3B, Additional file 4). These results suggested that both *tco89Δ* and H3K37A might be functionally related in their effects on cell-cycle regulation. We next repeated these cell-cycle experiments using wild-type yeast and H3K37A, H3K37R, and H3K37Q mutants to determine whether restoring positive charge at this position (H3K37R) or mimicking acetylation (H3K37Q) would restore a normal cell-cycle response. These experiments demonstrate that both H3K37R and H3K37Q rescued the cell-cycle defects (Figure 3C, Additional file 5) as well as growth on rapamycin plates (Figure 3D). Although H3K37 can be monomethylated or acetylated [39,40], it is unlikely that loss of histone modifications at this position causes the rapamycin phenotype, since both H3K37R and H3K37Q rescue growth on rapamycin.

Disruption of the TORC1 activating complex, EGO, or a *tco89Δ* results in an inability of cells to re-enter the cell cycle and proliferate after transient rapamycin treatment or nutrient starvation [9,41]. Owing to the similar effects on cell-cycle regulation between H3K37A and *tco89Δ* demonstrated above, we next compared the effects of transient rapamycin treatment on H3K37A relative to *tco89Δ*. Initially, equal numbers of log phase H3WT, H3K37A, wild-type, and *tco89Δ* cells were spotted to control YPD plates. The remaining cultures were then treated with 25 nM (sub-inhibitory) or 200 nM (inhibitory) rapamycin for 5.5 hours before washing and spotting to YPD plates to examine their ability to resume growth. After 5.5 hours, the H3K37A mutant exhibited only minor growth defects when treated with 25 nM rapamycin but had more pronounced growth defects when treated with 200 nM (Figure 3E). The *tco89Δ* mutant, however, was almost completely growth-impaired after transient treatment with 25 nM rapamycin, while growth was completely prevented by 200 nM rapamycin (Figure 3E). These experiments were repeated with the rapamycin treatment time extended to 24 hours. Under these conditions, H3K37A growth was significantly impaired after 24 hours with 25 nM rapamycin; this was further exacerbated by 200 nM rapamycin treatment (Figure 3F). These results demonstrate that the H3K37A mutation negatively affects cell-cycle progression and cell growth upon transient rapamycin treatment in a manner dependent on both the severity and length of time that TORC1 activity is impaired. These effects are similar, albeit not as severe, as those detected in *tco89Δ* under identical conditions.

### Limiting TORC1 activity in H3K37A results in necrosis and is functionally linked to the disruption of high mobility group (HMG) protein binding to chromatin

**Figure 4:**
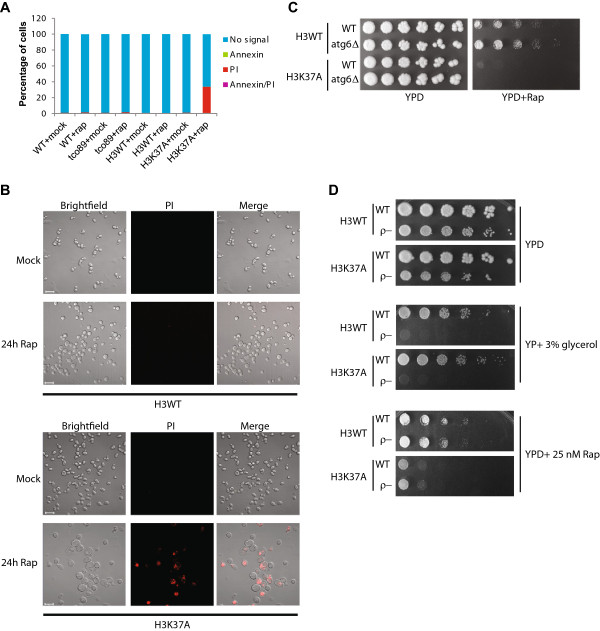
**Limited TORC1 signaling in the H3K37A mutant selectively induces necrosis. (A)** Wild-type (BY4741), *tco89Δ*, H3WT, and H3K37A cells were grown to log phase before mock treating or treating with 25 nM rapamycin for 24 hours. Viability was determined by staining cells with Annexin V and propidium iodide and analyzing by flow cytometry. **(B)** Mock-treated and rapamycin-treated H3WT and H3K37A mutants from **(A)** were visualized by confocal microscopy. Scale bar represents 10 μm. **(C)** H3WT and H3K37A mutants were combined with an *atg6Δ* to prevent autophagy and then spotted to control YPD or YPD + 25 nM rapamycin plates. Plates were incubated at 30°C for 4 days. **(D)** H3WT, H3K37A, and the mitochondrial-deficient (ρ-) derivative strains from each background were spotted to control YPD, YP containing 3% glycerol and YPD containing 25 nM rapamycin plates. The YPD and YP + 3% glycerol plates were incubated at 30°C for 3 days while the YPD + 25 nM Rap plates were incubated at 30°C for 7 days. PI, propidium iodide; rap, rapamycin.

The inability of *tco89Δ* mutants to resume growth after transient TORC1 inhibition has been attributed to a permanent exit from the cell cycle without a loss of viability [9]. Given the similar phenotypes of *tco89Δ* and H3K37A, we next tested whether H3K37A also maintained viability after rapamycin treatment. Eukaryotic cell-death pathways can be defined by staining cells with Annexin V (which indicates apoptotic cells) and propidium iodide (PI) (which indicates necrotic cells). To confirm that we could reliably detect both forms of cell death, we subjected wild-type cells to either a mock treatment or treatment with 80 mM acetic acid for three hours, a condition known to induce both apoptosis and necrosis [42]. While negligible Annexin V and PI staining occurred in the mock-treated samples, we readily detected both Annexin V and PI positive cells in the acetic acid treated samples (Additional file 6), thus demonstrating the reliability of this approach in analyzing cell-death responses. We next repeated the 25 nM rapamycin treatment of wild-type, *tco89Δ*, H3WT, and H3K37A strains for 24 hours, stained the cells with Annexin V and PI and analyzed them using flow cytometry. The wild-type, *tco89Δ*, and H3WT cells did not exhibit significant staining with either Annexin V or PI before or after rapamycin treatment (Figure 4A). These results confirm that while *tco89Δ* mutants are permanently growth arrested after TORC1 inhibition they do maintain viability [11]. However, the H3K37A mutant exhibited a significant increase in PI positive, but not Annexin V positive, cells, thus indicating that the cells were dying through a nonapoptotic pathway (Figure 4A). Analysis of H3WT and H3K37A by confocal microscopy revealed that H3K37A rapamycin-treated cells were considerably larger and more swollen than the mock-treated H3K37A cells or H3WT cells in either condition (Figure 4B).

Both autophagy, which is normally suppressed by TORC1, and mitochondrial dysfunction can be a source of nonapoptotic cell death [43]. We reasoned that if H3K37A mutants were dying as the result of an autophagic mechanism, then preventing autophagy by deleting the gene coding for the essential autophagy regulator, Atg6, would rescue H3K37A rapamycin growth. To test this, we engineered *atg6Δ* in both the H3WT and H3K37A backgrounds and spotted cells to control and rapamycin plates. Both the H3WT and H3WT *atg6Δ* grew comparably on rapamycin plates yet neither H3K37A nor the H3K37A *atg6Δ* grew (Figure 4C), demonstrating that the H3K37A mutants were dying through a necrotic, but not autophagic, cell-death pathway. Furthermore, induction of necrosis probably does not require functional mitochondria, as mitochondrial-deficient H3K37A cells (ρ-) also failed to rescue growth on rapamycin (Figure 4D). Therefore, H3K37A mutants undergo a mitochondrial-independent necrotic cell-death response under conditions of limited TORC1 signaling.

**Figure 5:**
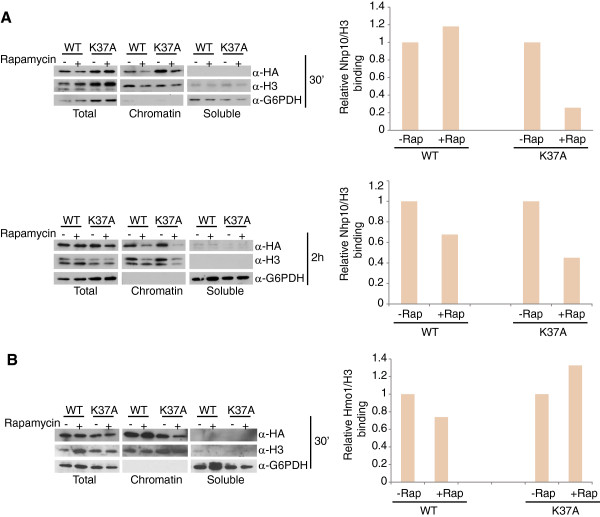
**Reduced TORC1 signaling and the H3K37A mutation destabilizes Nhp10 binding to chromatin. (A)** H3WT and H3K37A strains expressing Nhp10-6XHA were grown to log phase before mock treating or treating with 25 nM rapamycin for 30 minutes (upper panels) or 2 hours (lower panels). Samples were fractionated into soluble and chromatin-bound proteins and 30 μg of each was immunoblotted with the indicated antibody. Each set of immunoblots was exposed for the same length of time. Chromatin-bound Nhp10 and histone H3 were quantified with ImageJ software and expressed as a ratio of Nhp10/histone H3. The rapamycin samples were normalized to the mock controls, which were set to a value of 1. **(B)** As (A), except strains expressed Hmo1-6XHA. Only the 30-minute rapamycin treatment is shown, since Hmo1 protein levels decrease significantly by 2 hours post-rapamycin treatment. Rap, rapamycin; WT, wild-type.

H3K37 makes a direct contact with the mammalian high mobility group box 1 (HMGB1) protein in *in vitro* binding assays [44]. We speculated that rapamycin-induced necrosis in H3K37A may be functionally linked to decreased chromatin association of yeast HMG proteins. Yeast have seven HMG proteins, six of which (Nhp6a, Nhp6b, Abf2, Ixr1, and Hmo1) share significant homology to HMGB1 as determined by BLAST analysis of human HMGB1 against the *Saccharomyces* genome database [45,46]. To determine whether the H3K37A mutation impacts HMG chromatin association, we genomically tagged two distinct model HMG proteins, Nhp10 and Hmo1, with a 6XHA epitope in the H3WT and H3K37A backgrounds. We chose to examine Nhp10 because it is a component of a chromatin-remodeling complex, INO80, that has broad roles in transcription, DNA repair, and DNA replication [47,48]. As such, altered binding of Nhp10 by TORC1-regulated chromatin changes has the potential to affect a variety of genomic processes that could be functionally linked to the H3K37A phenotype. Hmo1 was chosen because this HMG protein regulates TORC1-dependent gene transcription [49-51] and its chromatin binding is affected by TORC1-regulated histone acetylation [36]. These strains were grown to log phase and either mock treated or treated with 25 nM rapamycin for 30 minutes or 2 hours before processing samples into soluble and chromatin-bound fractions. These fractions were then analyzed by α-HA immunoblots to detect the presence of Nhp10 or Hmo1. Under these conditions, rapamycin treatment in H3WT had no effect on Nhp10 chromatin association after 30 minutes but did modestly decrease Nhp10 binding after two hours (Figure 5A). Intriguingly, TORC1 inhibition in H3K37A resulted in decreased Nhp10 chromatin association at both 30 minutes and 2 hours (Figure 5A). Identical experiments in the Hmo1 background revealed a modest decrease in Hmo1 chromatin association in the H3WT background at 30 minutes while the H3K37A mutation did not significantly reduce Hmo1 binding under these conditions (Figure 5B). The difference in Hmo1 binding under these conditions in the H3WT and H3K37A is not completely understood. However, one possible explanation could be that the H3K37A mutation creates significant selective pressure for cells to maintain Hmo1 bound to TORC1 regulated genes during short periods of TORC1 inhibition and that prolonged TORC1 inhibition may ultimately result in decreased Hmo1 binding. Unfortunately, Hmo1 protein levels decrease upon longer rapamycin treatment (data not shown) so we were not able to ascertain Hmo1 chromatin association reliably at these later time points. Membranes were also probed for histone H3 and the cytoplasmic protein glyceraldehyde-6-phosphate dehydrogenase (G6PDH) to control for the quality of the soluble and chromatin fractions. The bulk of the cytoplasmic protein G6PDH was in the soluble fraction, while histone H3 was detected almost exclusively in the chromatin fraction (Figure 5A and B). We note that in these experiments we consistently detected both a full-length histone H3 band and a significant degradation product. Whether this breakdown product is a consequence of the fractionation protocol or is biologically relevant is unknown.

**Figure 6:**
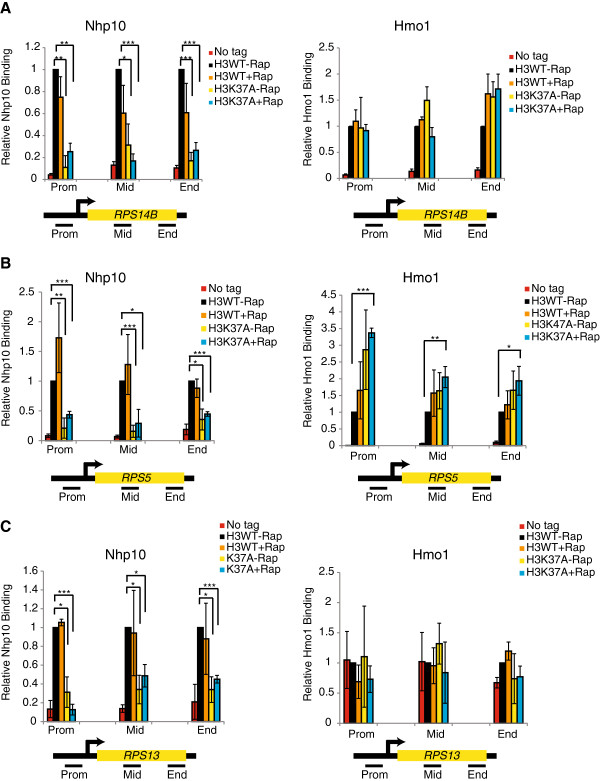
**The H3K37A mutation selectively disrupts Nhp10, but not Hmo1, binding to ribosomal protein genes.** The indicated strains were either mock treated or treated with 25 nM rapamycin for 30 minutes before performing chromatin immunoprecipitation analysis for either Nhp10 and Hmo1 binding at **(A)***RPS14B*, **(B)***RPS5*, **(C)***RPS13*. The relative positions of primers used in qPCR are indicated below each graph. The average and standard deviations of 3 (Nhp10) or 4 (Hmo1) independent experiments are plotted with significance determined by Student’s *t* test. *P<0.05; **P<0.01; ***P<0.005. Rap, rapamycin.

Because these chromatin association experiments measure global HMG binding and are more susceptible to experimental variability, we chose next to perform chromatin immunoprecipitation at multiple ribosomal protein genes to better quantify these effects on a gene-specific basis. Relative to the no-tag control strain, Nhp10 binding was significantly enriched at *RPS14B*, *RPS5* and *RPS13* (Figure 6A-C). Intriguingly, Nhp10 binding was reduced in the H3K37A mutant even before TORC1 inhibition and was not decreased further after a 30-minute rapamycin treatment (Figure 6A-C). These results demonstrate that the H3K37A mutation, even in the context of normal TORC1 signaling, is capable of significantly impeding Nhp10 binding at specific TORC1-regulated target genes. While Hmo1 was enriched at *RPS14B,* its binding was not significantly affected in either the wild-type strain or H3K37A mutant before or after rapamycin treatment (Figure 6A). Intriguingly, at *RPS5*, we detected a significant increase in Hmo1 binding throughout the gene body in the H3K37A rapamycin-treated samples (Figure 6B), which is consistent with our chromatin association assays (Figure 5B). We also examined binding of both Nhp10 and Hmo1 to *RPS13*, which was previously determined not to be a Hmo1-regulated gene [49]. At *RPS13*, Nhp10 binding in the H3K37A mutant is similarly disrupted, as seen for *RPS14B* and *RPS5*, whereas Hmo1 is not significantly enriched relative to the negative control (Figure 6C). Therefore, Nhp10 gene-specific binding is disrupted in the H3K37A mutant even in the context of normal TORC1 signaling whereas Hmo1 binding is not significantly impeded.

**Figure 7:**
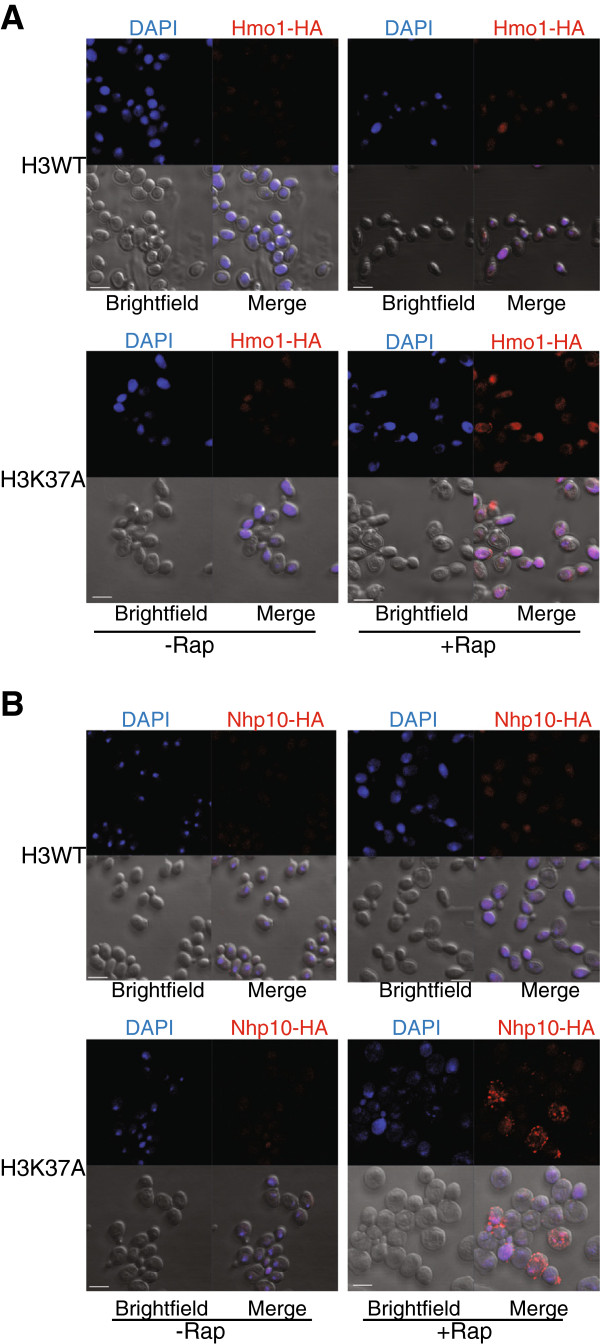
**The effect of TORC1 inhibition on Hmo1 and Nhp10 localization in H3WT and H3K37A. (A)** H3WT and H3K37A strains expressing Hmo1-6XHA were mock treated or treated with 25 nM rapamycin for 30 minutes before staining with rhodamine-conjugated α-HA antibody and DAPI. **(B)** As **(A)** except Nhp10-6XHA expressing cells were treated with 25 nM rapamycin for two hours. Cells were analyzed by confocal microscopy as described in the Methods and the data are representative of four independent experiments. Scale bar indicates 5 μm. Rap, rapamycin.

To analyze further the impact that H3K37A and TORC1 inhibition has on Hmo1 and Nhp10, we performed indirect immunofluorescence against these HA-tagged factors using the rapamycin treatment conditions described previously. In the mock-treated samples, Hmo1 in both the H3WT and H3K37A backgrounds exhibited a diffuse staining, which became more condensed after 30-minute rapamycin treatment (Figure 7A). This staining was specific for Hmo1 and Nhp10 (Figure 7B), as cells not expressing these HA-tagged factors exhibited no staining (Additional file 7). Under these conditions, Hmo1 cellular localization does not appear to be significantly impacted by the H3K37A mutation either before or after rapamycin treatment (Figure 7A). However, since Hmo1 levels decrease upon longer rapamycin treatment, later time points could not be reliably examined. As such, we cannot exclude the possibility that Hmo1 localization at later time points is affected by reduced TORC1 signaling.

Staining for Nhp10 demonstrated that, like Hmo1, it is broadly distributed in both H3WT and H3K37A mutants in the mock-treated samples. After 2-hour rapamycin treatment, Nhp10 became more condensed in the H3WT background in a manner similar to that seen for Hmo1 (Figure 7B). Interestingly, in the H3K37A mutant, Nhp10 distribution also becomes more condensed. However, in some H3K37A cells, punctate foci of intense Nhp10 staining were also detected that did not completely localize to the nucleus, suggesting that at least some Nhp10 might be moving into the cytoplasm (Figure 7B, lower right merged panel). This is specific for H3K37A, as rapamycin treatment did not induce these Nhp10 foci in the H3WT background (Figure 7B, compare upper right and lower right panels). The H3K37A rapamycin-treated cells were larger than the matched H3WT cells; this finding is consistent with the effect that longer-term (24-hour) rapamycin treatment had on H3K37A (Figure 4B). Overall, these results demonstrate that TORC1 signaling is required for steady-state global chromatin binding by Nhp10, and to a minor extent, Hmo1, and that the H3K37A mutation alone can negatively affect Nhp10 binding to specific TORC1-regulated genes. These results also suggest that decreased TORC1 signaling in H3K37A affects the cellular distribution of Nhp10, resulting in at least some Nhp10 relocating to the cytoplasm.

### Deregulation of HMG proteins actively induces necrosis

**Figure 8:**
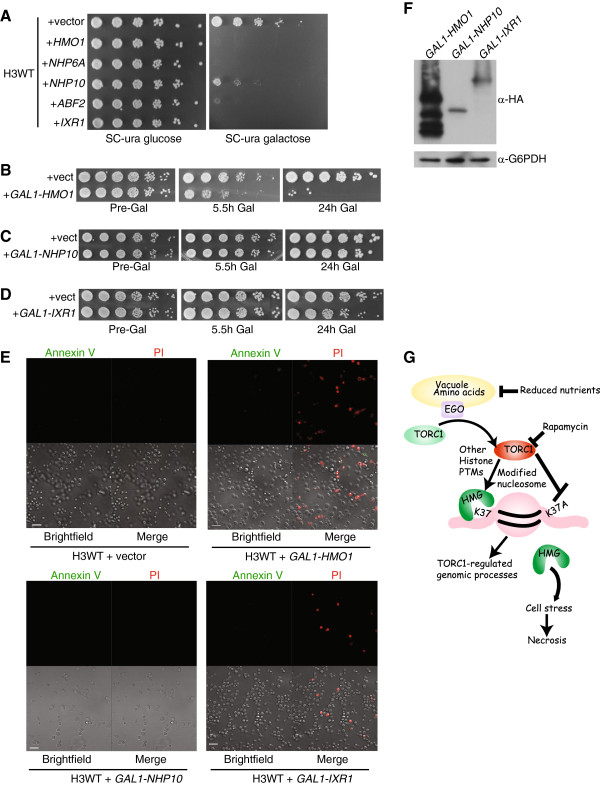
**Deregulation of HMG proteins induces necrosis. (A)** H3WT cells were transformed with control vector or a vector expressing one of the five indicated yeast HMG genes from the *GAL1* promoter. Equal numbers of cells were 5-fold serially diluted and spotted to SC-uracil plates containing glucose (repressive) or SC-uracil galactose (activating) media and incubated at 30°C for 4 days. (B-D) Cells containing control vector and either the galactose-inducible Hmo1 (B), Nhp10 (C) or Ixr1 (D) expression vectors were grown to log phase in raffinose media before serially diluting 5-fold and spotting to SC-uracil glucose plates. The remaining cultures were treated with 2% galactose for the indicated times before washing and spotting as described. Plates were incubated for 3 days at 30°C. **(E)** H3WT cells carrying control, *GAL1-HMO1*, *GAL1-NHP10*, or *GAL1-IXR1* expression vectors were grown to log phase in SC-uracil raffinose media, induced with 2% galactose for 24 hours, stained with Annexin V and PI, and then analyzed by confocal microscopy. Scale bar indicates 10 μm. **(F)** The strains from **(B-D)** were induced with 2% galactose for 1 hour before preparing whole-cell extracts. 30 μg of extract was resolved by SDS-PAGE and immunoblotted with α-HA antibody to detect the HA-tagged Hmo1, Nhp10 and Ixr1. **(G)** Model for TORC1 signaling and regulation of HMG chromatin association. Details in the text. Gal, galactose; PI, propidium iodide; ura, uracil, vect, vector.

Relocation of HMGB1 and HMGB1 orthologs from the nucleus to the cytoplasm has been used as an indicator of necrosis in eukaryotes [52,53]. Our data implicate TORC1 signaling as a candidate regulator of chromatin binding for at least Nhp10 and perhaps other untested HMG proteins as well. We considered the possibility that decreased TORC1 activity might act in combination with H3K37A to affect the chromatin association of one or more HMG proteins and that this might actually constitute an initiating signal for necrosis. To begin testing this possibility, we transformed H3WT with either a control vector or individual vectors expressing five (*HMO1*, *NHP6A*, *NHP10*, *ABF2* or *IXR1*) of the seven yeast HMG proteins from the galactose-inducible *GAL1* promoter [54]. We reasoned that high-level expression of individual HMGs from the *GAL1* promoter would generate at least some non-chromatin-bound HMG protein. If non-chromatin-bound HMGs can initiate necrosis, then we would expect significantly impaired growth responses when these factors are overexpressed. Transformed cells were spotted to selective media containing glucose (to repress) or galactose (to induce) expression of the indicated HMG factors and incubated for three days. While all cell populations grew comparably on glucose media, only cells carrying the control vector grew well on galactose media. Expression of the HMG genes *HMO1*, *NHP6A*, *ABF2*, or *IXR1* resulted in no growth and expression of *NHP10* resulted in very poor growth (Figure 8A). We selected Hmo1, Ixr1, and Nhp10 for further analysis, since Hmo1- and Ixr1-overexpressing cells failed to grow whereas Nhp10-overexpressing cells grew, albeit poorly. We next determined whether transient galactose induction followed by glucose repression would significantly impair cell growth. Wild-type cells carrying control vector or either *GAL1-HMO1*, *GAL1-NHP10*, or *GAL1-IXR1* were grown to log phase in selective media containing raffinose and equal numbers of cells were spotted to control media (pre-gal samples). The remaining cultures were treated with 2% galactose for 5.5 or 24 hours and then washed and spotted to glucose media and allowed to recover for three days. Under these conditions, transient induction of *HMO1* for 5.5 hours significantly impaired growth, an effect that was dramatically exacerbated after 24 hours induction (Figure 8B). Although constitutive *NHP10* expression resulted in poor growth, transient *NHP10* expression did not impair growth after either 5.5 or 24 hours (Figure 8C). Transient induction of *IXR1* for 5.5 hours did not significantly impair growth but after 24 hours *IXR1* expression resulted in modest growth defects (Figure 8D). Robust expression of Hmo1, Ixr1, and Nhp10 protein was detected after a one-hour galactose induction (Figure 8F), thus making these effects unlikely to be due to significant differences in expression of these HMGs from the *GAL1* promoter.

To determine whether the growth defects in the Hmo1- and Ixr1-overexpressing cells were due to loss of cell viability, H3WT cells carrying either control, *GAL1-HMO1*, *GAL1-NHP10*, or *GAL1-IXR1* expression vectors were induced with galactose for 24 hours. Cells were then stained with Annexin V and PI and analyzed by confocal microscopy. Neither the control vector nor the *GAL1-NHP10* vector resulted in significant Annexin V or PI staining under these conditions (Figure 8E); this result is consistent with the lack of significant growth defects detected in cells transiently overexpressing Nhp10 (Figure 8C). Both Hmo1 and Ixr1 overexpression resulted in significant numbers of PI positive cells, with Hmo1 overexpression causing a greater necrotic response than Ixr1 (Figure 8E). These results are consistent with the overall effects their transient expression had on cell growth (compare Figure 8B and 8D). Intriguingly, while significant necrosis was detected in both Hmo1- and Ixr1-expressing cells, no increase in apoptotic cells was observed. These results indicate that the growth defects detected in Hmo1- or Ixr1-overexpressing cells are due to loss of viability caused by necrosis whereas the poor growth phenotype detected in Nhp10-expressing cells (Figure 8A) is independent of cell death. Although both Hmo1 and Ixr1 overexpression induced necrosis, neither HMG alone caused the same enlarged, swollen cell phenotype evident in the H3K37A rapamycin cells (compare Figure 4B and Figure 8E). Therefore, the H3K37A mutation probably affects chromatin binding by multiple HMG proteins during conditions of reduced TORC1 activity to cause these more severe morphological effects. Overall, these results implicate a role for TORC1 signaling in promoting the chromatin binding of HMG proteins and provide evidence that HMG deregulation can actively initiate a necrotic cell-death response.

## Discussion

Environmental nutrients affect chromatin regulation to alter epigenetic processes controlling growth, proliferation and developmental potential of eukaryotic cells [1,2]. The mechanisms underlying this process remain poorly characterized, yet it is essential that they are addressed, as they have profound implications for many diseases, including cancer, diabetes, and cardiovascular disease [1,2]. We have begun to address this issue by utilizing the TORC1 pathway as a model, since it is a critical transducer of nutrient information in eukaryotes and is deregulated in numerous diseases [55]. The rationale for our approach was predicated on the idea that histone residues functionally linked to TORC1, and as an extension those chromatin pathways involving these residues, would exhibit altered growth responses upon rapamycin treatment. Our genetic screens have identified a number of histone residues, predominantly on histone H3, linked to TORC1 via this approach. Many of these residues are known sites of histone post-translational modification or are contact residues for non-histone chromatin components, so we believe these data will serve as an initiation point for delineating how TORC1 regulates chromatin structure and function. Since most of these histone residues are conserved from yeast to man, our results suggest the possibility that TORC1 may utilize a core group of evolutionarily conserved chromatin pathways that includes a predominant role for histone H3.

In this study, we examined a subset of histone H3 mutants exhibiting both sensitive (H3K14A and H3K37A) and resistant (H3T3A, H3Q5A, and H3S57A) rapamycin phenotypes to understand their specific functions in the TORC1 pathway. Intriguingly, while H3K14A and H3K37A were both sensitive to rapamycin, only H3K37A exhibited sensitivity to the less specific TORC1 inhibitor caffeine, thus demonstrating an absolutely essential role for H3K37 under conditions of even mild TORC1 perturbation. Because Sch9 activation could rescue the H3K14A but not the H3K37A rapamycin phenotype, these experiments demonstrate that the role of H3K37 in the TORC1 pathway is independent of the downstream TORC1 effector kinase Sch9. Our data further suggest that the essential nature of H3K37 is highly specific for TORC1, since H3K37A was not affected by reduced Ras/PKA signaling.

Except for H3K37A, neither the gene expression analyses nor the cell-cycle studies of the remaining histone mutants resulted in clear outcomes corresponding to their rapamycin phenotypes. These results suggest that their contributions to the TORC1 phenotype are likely to be complex in nature and cannot be ascribed simply to altered ribosomal transcription or dysfunctional cell-cycle dynamics. Interestingly, these studies did reveal previously unappreciated roles for H3T3 and H3Q5 in TORC1-regulated gene expression. Specifically, these residues were demonstrated to be critical for *RPS14B* expression in nutrient-replete conditions. The decreased *RPS14B* expression in H3T3A and H3Q5A were unlikely to be due to loss of H3K4 methylation, as has been proposed [56], since H3K4A increased *RPS14B* expression. Recently, a study demonstrated that H3K4me3, which normally demarcates actively transcribed genes, can behave as a repressive chromatin modification specifically for RP gene transcription under conditions of stress including that induced by rapamycin treatment [57]. Therefore, our results suggest that H3T3A and H3Q5A may actually impact TORC1-regulated RP gene transcription by either disrupting H3K4me3 demethylation or through mechanisms independent of H3K4 post-translational modification altogether. Furthermore, H3Q5 has a positive role in 5S rRNA expression independent of H3T3 and H3K4me3 and thus may contribute uniquely to RNA Pol III-dependent transcription.

Under nutrient-replete conditions, the minor increase in ITS1-containing rRNA levels in H3K37A is likely to be due to a disruption in rDNA chromatin structure required for 35S rRNA co-transcriptional processing. This effect could be similar in nature to that originally characterized by our laboratory for mutants of the H3K56ac pathway [36]. However, the remainder of the rapamycin-induced gene expression and cell-cycle effects in H3K37A are probably indirectly caused by the induction of cell death upon TORC1 inhibition. This cell-death response in H3K37A is clearly distinct from the severe growth defect occurring in *tco89Δ* after TORC1 inhibition, since this mutant maintains viability but is unable to re-enter the cell cycle upon rapamycin removal. The lack of significant Annexin V staining and the inability of the *atg6Δ* to rescue H3K37A growth on rapamycin implicate necrosis as the cause of rapamycin-induced cell death in H3K37A. While H3K37 can be monomethylated or acetylated [39,40], loss of either of these modifications does not explain the cell-death response, since both H3K37R and H3K37Q mutations rescue growth on rapamycin. Instead, we believe the H3K37A mutation probably disrupts an HMG contact residue [44], which alters the chromatin association of a subset of HMG proteins. This effect would then be further exacerbated by changes to chromatin that occur on TORC1 inhibition. These changes might include decreased H3K56ac, since TORC1 regulates this histone modification and disruption of this chromatin pathway results in similar cell-death phenotypes [36,58]. Alternatively, other histone post-translational modifications, particularly those occurring on the histone H3 and H4 residues identified in this study, could be perturbed under these conditions. These chromatin changes could act in concert with the H3K37A mutation to impede HMG chromatin binding further. Consistent with this concept, we provide evidence that Nhp10 chromatin binding is impaired by both decreased TORC1 activity and the H3K37A mutation. Since Nhp10 and select other HMG proteins are constituents of ATP-dependent chromatin-remodeling and histone chaperone complexes [47,59], regulating their chromatin association by TORC1 signaling could allow cells to adjust their transcriptional programs quickly in response to a rapidly changing nutrient environment.

Our data demonstrate a significant role for TORC1 and H3K37 in regulating chromatin binding by Nhp10. However, Nhp10 overexpression was insufficient to induce necrosis, whereas both Hmo1 and Ixr1 overexpression could induce robust necrosis. Intriguingly, neither Hmo1 nor Ixr1 overexpression fully recapitulated the effects that TORC1 inhibition had in the H3K37A background. In total, our data suggest a speculative model by which severe nutrient deprivation significantly reduces TORC1 activity in wild-type cells, resulting in decreased chromatin binding by multiple HMG proteins simultaneously. In the H3K37A background, the chromatin binding of a subset of HMG proteins is already reduced, thus making this mutant even more sensitive to minor perturbations in TORC1 activity (Figure 8G). HMG chromatin dissociation could then alter global gene transcription or lead to increased localization of HMGs to the cytoplasm where they would disrupt signaling pathways or organelles (perhaps vacuoles/lysosomes) essential for maintaining cell homeostasis [60-63]. Which HMG, or combination of HMGs, evicted from chromatin is necessary to induce necrosis and the mechanisms underlying this process will need to be addressed in future studies. Intriguingly, if correct, this model would directly implicate decreased HMG chromatin binding as a cause of necrosis instead of a consequence of necrotic cell death, as previously described [52,64]. This concept would be consistent with previous studies in multiple models demonstrating that necrosis is an ancient, genetically programmed cell-death pathway distinct from apoptosis and sensitive to specific stress events [43]. Overall, this study provides a foundation for determining how environmental nutrient signaling through TORC1 affects epigenetic processes. It also suggests a previously unappreciated role for chromatin as an HMG binding platform that suppresses necrosis during periods of limited TORC1 signaling.

## Conclusions

These studies provide the foundations for delineating how environmental nutrients signal through the TORC1 complex to mediate effects on the epigenome. Specifically, the individual histone H3 and H4 residues functionally interacting with the TORC1 pathway have been identified. Since a number of these residues are sites of post-translational modification, these residues could be the endpoint of chromatin pathways regulated by TORC1 to affect gene transcription and chromosome structure necessary for cell growth and proliferation. Excitingly, these results also reveal an absolutely essential and unappreciated role for histone H3K37 in suppressing necrosis during periods of limited TORC1 activity that is linked to its role in promoting HMG protein binding to chromatin. Our results also provide direct evidence that HMG protein deregulation can be a stimulus for inducing necrosis, unlike previous studies that correlated the release of HMG proteins from chromatin as a consequence of necrosis. Overall, these studies provide novel new insights into TORC1 interactions with the epigenome and suggest that one function of the chromatin fiber is to bind HMG proteins under conditions of decreased TORC1 signaling to prevent cell death by necrosis.

## Methods

### Yeast media, plasmids, cell growth conditions, and chemicals

All of the yeast histone mutants used in this study are from the histone H3/H4 library derived by Dai *et al*. [22] and purchased from Open Biosystems. Specific strains and plasmids used throughout the study are listed in Additional file 8. Strains YNL372 (H3WT), YNL401 (H3T3A), YNL357 (H3Q5A), YNL352 (H3K14A), YNL403 (H3K37A), and YNL374 (H3S57A) used in our detailed analyses are derivative strains from the histone library that were made Ura- by growth on 5-FOA media to select for those clones that recombined out the *URA3* marker. Unless otherwise stated, cells were grown in nutrient-rich YPD (2% peptone, 1% yeast extract, 2% dextrose) or YPD containing 25 nM rapamycin. For growth on selective synthetic complete medium (SC), SC containing 1 g/l of glutamic acid as a nitrogen donor and lacking the appropriate dropout component was utilized. To generate ρ- mutants, H3WT and H3K37A strains were cultured through two successive rounds of growth in SC medium containing 25 μg/ml ethidium bromide. Single colonies were then picked and the mitochondrial deficiency phenotype was confirmed by lack of growth on a nonfermentable carbon source (YP + 3% glycerol). All yeast growth media and reagents were purchased from Research Products International or US Biologicals. All chemicals used in these experiments were purchased from Fisher Scientific or Sigma-Aldrich.

The rapamycin screens of the histone library were performed by growing wild-type yeast and the indicated mutants overnight in YPD at 30°C with shaking. The following day, equal cell numbers were 5-fold serially diluted and replica spotted to control YPD or YPD containing 25 nM rapamycin plates and incubated at 30°C. Growth was analyzed every day and photographs were typically taken between three and six days. The results were scored using the methodology described in Rizzardi *et al*. [65]. Briefly, those mutants exhibiting synthetic growth phenotypes relative to wild-type controls were scored numerically from 1 to 3, with 1 being approximately a 5-fold difference in growth and 3 being a 125-fold difference in growth. Mutants exhibiting growth defects were given negative values while mutants showing enhanced growth were given positive values. A value of 0 indicates no difference from the wild-type control strain. For the transient rapamycin treatment and recovery experiments, cells were initially grown to log phase. Equal numbers of each strain were removed, 5-fold serially diluted and then spotted to YPD plates. The remainder of each culture was treated with the specific concentration of rapamycin for the indicated time and then equal numbers of cells were gently pelleted, washed with sterile water, diluted, and spotted, as described. Pictures were taken after 3 days incubation at 30°C.

### RNA extraction, cDNA synthesis, and quantitative PCR (qPCR)

RNA was extracted using Tri-Reagent (Sigma-Aldrich) and bead beating with subsequent extensive digestion using DNase I to eliminate contaminating genomic DNA. 1 μg total RNA was then used in a cDNA synthesis reaction with random primers and the Im-Prom II cDNA synthesis kit (Promega, Inc.). cDNAs were resuspended to a final volume of 100 μl, diluted 1:5 and then used in qPCR with primers to the indicated genes. The gene-specific signals were normalized to the internal control gene *ACT1* using the formula 2^(*ACT1* Ct-Target Gene Ct)^. After normalization, the wild-type sample was set to a value of 1 and each mutant expressed relative to wild-type with statistical significance determined by Student’s *t* test. Primer sequences are available upon request.

### Whole-cell extract preparation, immunoblotting, and antibodies

Whole-cell extracts from log phase yeast cultures were prepared in protein extraction buffer (10 mM Tris, pH 8.0, 150 mM NaCl, 0.1% Nonidet P-40, 10% glycerol, 1 mM DTT) containing protease and phosphatase inhibitors by bead beating, as described previously [36]. For immunoblot analyses, 30 μg of whole-cell extracts were resolved by 10% SDS-PAGE and then transferred to polyvinylidene difluoride membrane before incubating with the indicated antibody. The α-HA antibody was purchased from Santa Cruz Biotechnology, Inc., the α-β actin antibody was purchased from Abcam, the α-G6PDH antibody was obtained from Sigma-Aldrich, and the α-H3 antibody was purchased from Active Motif. After incubation with the appropriate secondary antibody, chemiluminescence was performed using the Immobilon Chemiluminescent HRP system from Millipore and samples were detected by exposure to film.

### Chromatin association assay

The chromatin association assay is a combination of two previously published protocols [66,67]. Briefly, 100 ml of cells (OD_600_ ≈ 0.5) were collected and washed with protein solubilization buffer (PSB) (20 mM Tris-Cl pH 7.4, 2 mM EDTA, 100 mM NaCl, 10 mM β-mercaptoethanol) and then solubilization buffer (SB) (1 M sorbitol, 20mM Tris-Cl pH 7.4). Cell pellets were then incubated with 1 mg/ml Zymolyase 20T in SB buffer for 30 minutes to 1 hour at room temperature. The resulting spheroplasts were washed twice with 1 ml LB buffer (0.4 M sorbitol, 150 mM potassium acetate, 2 mM magnesium acetate, 20 mM PIPES, with the pH adjusted to 6.8 using potassium hydroxide) containing protease inhibitors (1 mM phenylmethanesulfonylfluoride, 10 μg/ml leupeptin, 10 μg/ml aprotinin, 10 μg/ml pepstatin) and then pelleted at 2000 rpm for 5 minutes at 4°C. Cell pellets were resuspended in 250 μl LB buffer plus 1% Triton X-100 containing protease inhibitors and incubated on ice with occasional mixing for 10 minutes. 125 μl of this sample was removed to use as the total fraction while the remainder of the lysate was centrifuged at 5000*g* for 15 minutes at 4°C. The supernatant (soluble protein fraction) was removed while the chromatin pellet was washed in 125 μl LB containing 1% Triton X-100 and protease inhibitors and then pelleted at 5,000*g* for 15 minutes at 4°C. The supernatant was discarded and the resulting chromatin fraction was resuspended in 125 μl LB buffer containing 1% Triton X-100 and protease inhibitors. The Bradford assay was used to normalize protein levels for the total, soluble, and chromatin-bound fractions and 30 μg of each was used for SDS-PAGE and immunoblot analysis. The levels of chromatin-bound HMG and histone H3 proteins were quantified by ImageJ analysis, expressed as a ratio of HMG/histone H3 and the rapamycin-treated sample then normalized to the mock-treated sample, which was set to a value of 1.

### Chromatin immunoprecipitation analysis

A no-tag control and the indicated H3 wild-type or H3K37A mutants carrying either Nhp10-6xHA or Hmo1-6XHA were grown in YPD to an OD_600_ = 1.0 to 1.5 before cross-linking for 15 minutes with 1% formaldehyde and then quenching with 125 mM glycine for 5 minutes. Chromatin extracts were prepared and immunoprecipitation was performed as described [36] using 500 μg of the chromatin extract and 1 μg of α-HA antibody. The immunoprecipitated chromatin, as well as 10% of input chromatin, was purified and eluted in 50 μl (for immunoprecipitation) or 100 μl (for inputs). Samples were then diluted 1:5 for immunoprecipitation and 1:100 for inputs before analyzing by real-time PCR. The enrichment was determined using the following formula: 2^(Input Ct-IP Ct)^. The sample of H3 wild-type without rapamycin treatment was set to a value of 1 and the remaining samples, including the no-tag control, was expressed relative to this sample. Primer sequences are available upon request.

### Flow cytometry and immunofluorescence analysis

The fluorescent dye SYTOX Green (Invitrogen, Inc.) was used to stain yeast DNA as previously described [68]. Samples were analyzed on a BD LSD II flow cytometer using Modfit software for data analysis at the UT flow cytometer facility. Immunofluorescence analysis was performed as follows. FITC-coupled Annexin V (ClonTech Laboratories, Inc.) and PI (MP Biomedical, LLC) were used to stain live cells, as previously described [69]. Briefly, yeast cells were stained in binding buffer (10 mM HEPES/NaOH, PH 7.4, 140 mM NaCl, 2.5 mM CaCl_2_) with Annexin V at a concentration of 1μg/ml and a PI concentration of 25 μg/ml for 20 to 30 minutes at room temperature. Slides were prepared by pouring an agarose cushion to prevent movement of the yeast cells and then analyzed using a 63X oil Olympus objective on a Zeiss LSM 700 confocal microscope with its own software to capture and analyze the images.

For the indirect immunofluorescence analysis of Hmo1 and Nhp10, cells were grown to log phase and then fixed with 70% ethanol for one hour. Cells were then washed three times with 5% BSA before staining with primary mouse α-HA antibody (Santa Cruz Biotechnology, sc-7392) at a 1:25 dilution (from a 1 mg/ml stock) overnight. Cells were then washed three times with 5% BSA and stained with secondary IgG goat anti-mouse antibody conjugated to rhodamine (Millipore, LOT: 219844) in a 1:25 dilution overnight. After washing three times with 5% BSA, cells were subsequently stained with 2 ug/ml DAPI (MP Biomedicals Lot No: M5816) for 30 minutes, washing steps were repeated and the samples were then analyzed by confocal microscopy.

## Abbreviations

BSA: Bovine serum albumin; G6PDH: Glyceraldehyde-6-phosphate dehydrogenase; HMG: High mobility group; PCR: Polymerase chain reaction; PI: Propidium iodide; PKA: Protein kinase A; qPCR: Quantitative polymerase chain reaction; SC: Synthetic complete medium; TOR: Target of rapamycin; TORC1: Target of rapamycin complex 1; TORC2: Target of rapamycin complex 2; YP: Yeast extract and peptone; YPD: Yeast extract, peptone, and dextrose.

## Competing interests

The authors declare they have no competing interests.

## Authors’ contributions

HC, JJW, AT, and RNL designed and performed the experiments. RNL, in collaboration with all the other authors, wrote the manuscript, which was approved by all of the authors.

## Supplementary Material

Additional file 1 Immunoblot analysis of TORC1 pathway mutants. Click here for file

Additional file 2 18S and 25S rRNA levels in histone H3 mutants. Click here for file

Additional file 3 Cell-cycle analysis of histone H3 mutants after rapamycin treatment. Click here for file

Additional file 4 **Cell-cycle analysis of wild-type and *tco89Δ* cells after rapamycin treatment.** Click here for file

Additional file 5 Cell-cycle analysis of H3WT, H3K37A, H3K37R, and H3K37Q after rapamycin treatment. Click here for file

Additional file 6 Acetic acid induction of apoptotic and necrotic cell death. Click here for file

Additional file 7 Control staining for HMG confocal analysis. Click here for file

Additional file 8 Yeast strains and plasmids. Click here for file
