# Supplementary material for: Target of rapamycin signaling regulates high mobility group protein association to chromatin, which functions to suppress necrotic cell death
Source: Epigenetics Chromatin. 2013 Sep 2;6:29. doi: 10.1186/1756-8935-6-29 (PMC3766136; doi:10.1186/1756-8935-6-29)
Supplement: Additional file 1 — Immunoblot analysis of TORC1 pathway mutants. [file 1756-8935-6-29-S1.pdf]

Additional Files

Additional File 1. Immunoblot analysis of TORC1 pathway mutants. (A) H3WT, H3K14A and H3K37A strains transformed with control vector (H3WT) or vector expressing the Tor1 hyperactive mutant, Tor1^{A1957} (A) or the Sch9^{2D3E} mutant (B). Cells were grown to log phase in the appropriate selection media and whole-cell extracts were prepared. 30 μg of extract was resolved by SDS-PAGE and immunoblotted with α-HA antibody before stripping and reprobing with α-actin as a loading control.

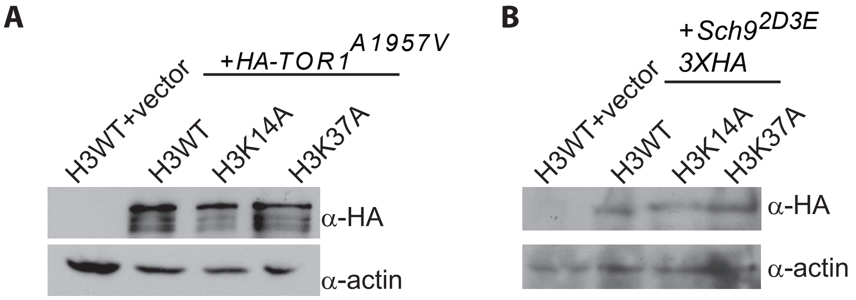

Additional File 1.
