# Supplementary material for: Target of rapamycin signaling regulates high mobility group protein association to chromatin, which functions to suppress necrotic cell death
Source: Epigenetics Chromatin. 2013 Sep 2;6:29. doi: 10.1186/1756-8935-6-29 (PMC3766136; doi:10.1186/1756-8935-6-29)
Supplement: Additional file 2 — 18S and 25S rRNA levels in histone H3 mutants. [file 1756-8935-6-29-S2.pdf]

Additional File 2. 18S and 25S rRNA levels in histone H3 mutants. The levels of 18S (A) and 25S (B) were analyzed as described in the figure legend for Figure 2. The data are the average and standard deviation of four independent experiments with significance determined by t-test. *-$p<0.05$.

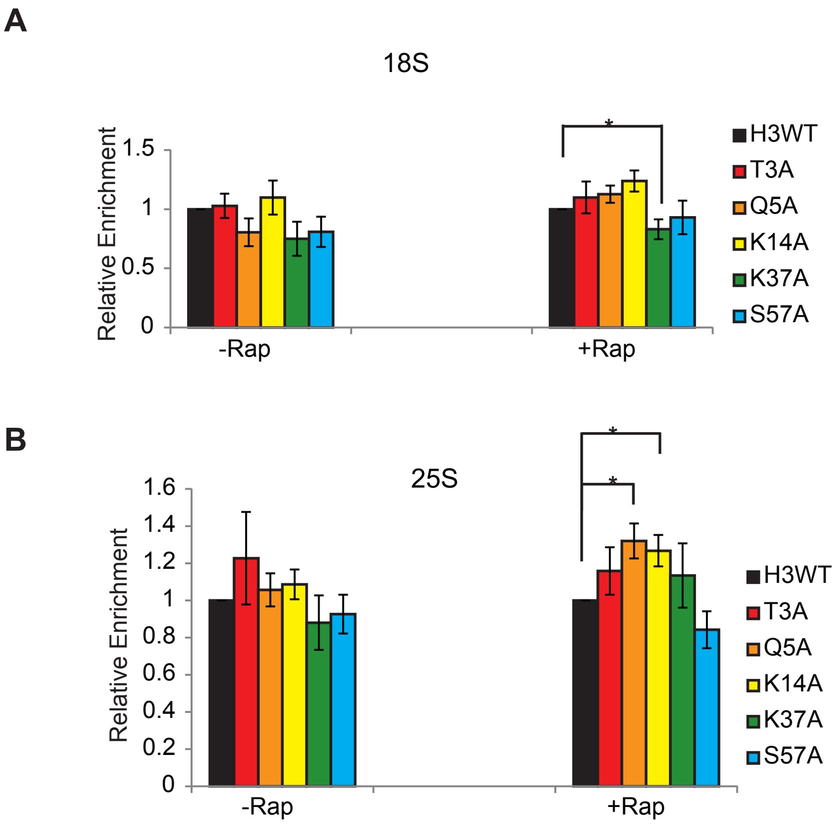

Additional Figure 2.
