# Supplementary material for: Target of rapamycin signaling regulates high mobility group protein association to chromatin, which functions to suppress necrotic cell death
Source: Epigenetics Chromatin. 2013 Sep 2;6:29. doi: 10.1186/1756-8935-6-29 (PMC3766136; doi:10.1186/1756-8935-6-29)
Supplement: Additional file 3 — Cell-cycle analysis of histone H3 mutants after rapamycin treatment. [file 1756-8935-6-29-S3.pdf]

Additional File 3. Cell-cycle analysis of histone H3 mutants after rapamycin treatment.

**Pre-Rap**	**% G1**	**% S**	**% G2**
H3WT	29.8	38.2	32
H3T3A	26.2	44	29.8
H3Q5A	28	34.2	37.8
H3K14A	27.9	29.6	42.5
H3K37A	31.2	33.1	35.7
H3S57A	29.6	40.8	29.6
**1.5h Rap**	**% G1**	**% S**	**% G2**
H3WT	62.7	12.6	24.7
H3T3A	58.7	9.3	32
H3Q5A	49.8	20.1	30.1
H3K14A	59.6	11.2	29.2
H3K37A	72.2	3	24.8
H3S57A	51	23	26
**4h Rap**	**% G1**	**% S**	**% G2**
H3WT	52.2	22.6	25.2
H3T3A	50.8	23.8	25.4
H3Q5A	49	23	28
H3K14A	52.5	28.7	18.8
H3K37A	67.4	8.1	24.5
H3S57A	54.7	19.9	25.5
