# Supplementary material for: Target of rapamycin signaling regulates high mobility group protein association to chromatin, which functions to suppress necrotic cell death
Source: Epigenetics Chromatin. 2013 Sep 2;6:29. doi: 10.1186/1756-8935-6-29 (PMC3766136; doi:10.1186/1756-8935-6-29)
Supplement: Additional file 4 — Cell-cycle analysis of wild-type and tco89Δ cells after rapamycin treatment. [file 1756-8935-6-29-S4.pdf]

Additional File 4. Cell-cycle analysis of wild-type and *tco89Δ* after rapamycin treatment.

**Wild-type**	**% G1**	**% S**	**% G2**
Pre-Rap	34.2	13.4	52.4
1.5h Rap	69	2.8	28.2
4h Rap	56.4	14.3	29.3
tco89Δ	**% G1**	**% S**	**% G2**
Pre-Rap	28.7	15.8	55.5
1.5h Rap	46.4	0	53.6
4h Rap	55	0	45
