# Supplementary material for: Target of rapamycin signaling regulates high mobility group protein association to chromatin, which functions to suppress necrotic cell death
Source: Epigenetics Chromatin. 2013 Sep 2;6:29. doi: 10.1186/1756-8935-6-29 (PMC3766136; doi:10.1186/1756-8935-6-29)
Supplement: Additional file 5 — Cell-cycle analysis of H3WT, H3K37A, H3K37R, and H3K37Q after rapamycin treatment. [file 1756-8935-6-29-S5.pdf]

Additional File 5. Cell-cycle analysis of H3WT, H3K37A, H3K37R and H3K37Q after rapamycin treatment.

**Pre-Rap**	**% G1**	**% S**	**% G2**
H3WT	25.4	46.2	28.4
H3K37A	31.1	35.5	33.4
H3K37R	28.9	40.6	30.5
H3K37Q	30.3	39.9	29.8
**1.5h Rap**	**% G1**	**% S**	**% G2**
H3WT	61.9	12.4	25.7
H3K37A	68.7	3.2	28.1
H3K37R	56.2	18.4	25.4
H3K37Q	66.7	11.9	21.3
**4h Rap**	**% G1**	**% S**	**% G2**
H3WT	54.3	21.5	24.2
H3K37A	63.2	5.8	31
H3K37R	53.2	21.8	25
H3K37Q	54.6	23.9	21.5
