# Supplementary material for: Target of rapamycin signaling regulates high mobility group protein association to chromatin, which functions to suppress necrotic cell death
Source: Epigenetics Chromatin. 2013 Sep 2;6:29. doi: 10.1186/1756-8935-6-29 (PMC3766136; doi:10.1186/1756-8935-6-29)
Supplement: Additional file 6 — Acetic acid induction of apoptotic and necrotic cell death. [file 1756-8935-6-29-S6.pdf]

Additional File 6. Acetic acid induction of apoptotic and necrotic cell death. H3WT cells were grown to log phase and then either mock-treated (A) or treated with 80 mM acetic acid (B) for three hours before staining with Annexin V and PI and confocal microscopy analysis. Scale bar represents 10 μm.

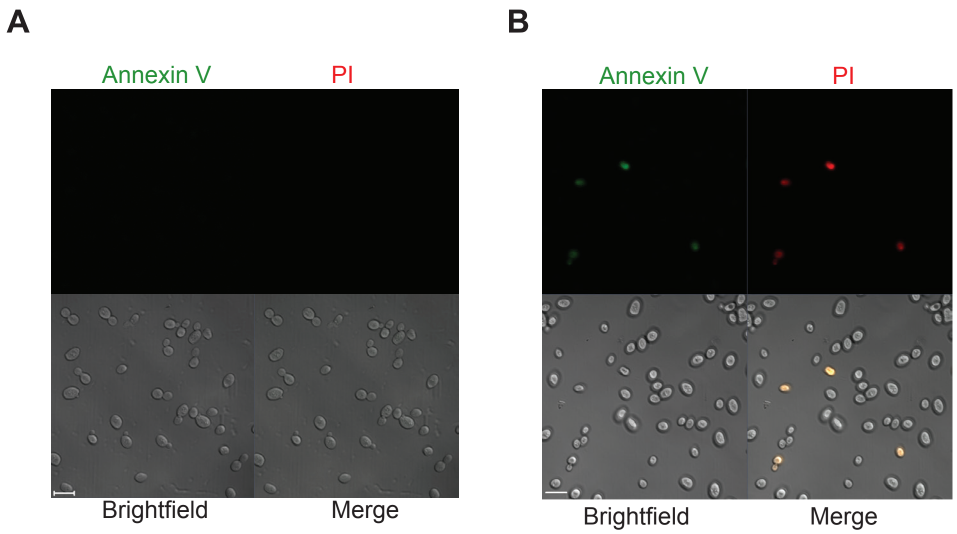

Additional File 6.
