# Supplementary material for: Target of rapamycin signaling regulates high mobility group protein association to chromatin, which functions to suppress necrotic cell death
Source: Epigenetics Chromatin. 2013 Sep 2;6:29. doi: 10.1186/1756-8935-6-29 (PMC3766136; doi:10.1186/1756-8935-6-29)
Supplement: Additional file 7 — Control staining for HMG confocal analysis. [file 1756-8935-6-29-S7.pdf]

Additional File 7. Control staining for HMG confocal analysis. A negative control H3WT strain lacking HA-tagged protein was stained as described in the Methods and analyzed by confocal microscopy to demonstrate the level of background HA staining. Scale bar represents 5 μm.

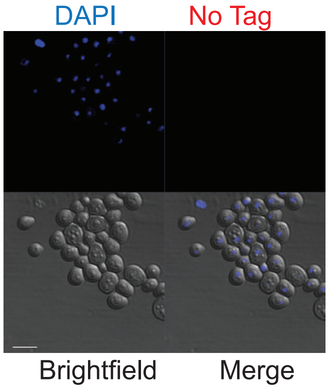

Additional File 7.
