# Supplementary material for: Target of rapamycin signaling regulates high mobility group protein association to chromatin, which functions to suppress necrotic cell death
Source: Epigenetics Chromatin. 2013 Sep 2;6:29. doi: 10.1186/1756-8935-6-29 (PMC3766136; doi:10.1186/1756-8935-6-29)
Supplement: Additional file 8 — Yeast strains and plasmids. [file 1756-8935-6-29-S8.pdf]

Additional File 8. Yeast strains and plasmids.

Yeast Strain	***Genotype***	***Reference/Source***
BY4741	MATa his3Δ1 leu2Δ met15Δ ura3Δ0	Open Biosystems
tco89Δ	BY4741; *tco89::KanMX*	Open Biosystems
YNL340	*MATa his3Δ200 leu2Δ0 lys2Δ0 trp1Δ63 ura3Δ0met15Δ0 can1::MFA1pr-HIS3 hht1-hhf1::NatMX4 hht2-hhf2::[HHTS-HHFS]*-ura3Δ H3WT HMO1-6XHA::HphNT1*	This study
YNL352 (H3K14A)	*MATa his3Δ200 leu2Δ0 lys2Δ0 trp1Δ63 ura3Δ0met15Δ0 can1::MFA1pr-HIS3 hht1-hhf1::NatMX4 hht2-hhf2::[HHTS-HHFS]*-ura3Δ*	This study
YNL357 (H3Q5A)	*MATa his3Δ200 leu2Δ0 lys2Δ0 trp1Δ63 ura3Δ0met15Δ0 can1::MFA1pr-HIS3 hht1-hhf1::NatMX4 hht2-hhf2::[HHTS-HHFS]*-ura3Δ*	This study
YNL372 (H3WT)	*MATa his3Δ200 leu2Δ0 lys2Δ0 trp1Δ63 ura3Δ0met15Δ0 can1::MFA1pr-HIS3 hht1-hhf1::NatMX4 hht2-hhf2::[HHTS-HHFS]*-ura3Δ*	This study
YNL374 (H3S57A)	*MATa his3Δ200 leu2Δ0 lys2Δ0 trp1Δ63 ura3Δ0met15Δ0 can1::MFA1pr-HIS3 hht1-hhf1::NatMX4 hht2-hhf2::[HHTS-HHFS]*-ura3Δ*	This study
YNL401 (H3T3A)	*MATa his3Δ200 leu2Δ0 lys2Δ0 trp1Δ63 ura3Δ0met15Δ0 can1::MFA1pr-HIS3 hht1-hhf1::NatMX4 hht2-hhf2::[HHTS-HHFS]*-ura3Δ*	This study
YNL403 (H3K37A)	*MATa his3Δ200 leu2Δ0 lys2Δ0 trp1Δ63 ura3Δ0met15Δ0 can1::MFA1pr-HIS3 hht1-hhf1::NatMX4 hht2-hhf2::[HHTS-HHFS]*-ura3Δ*	This study
YNL420	*MATa his3Δ200 leu2Δ0 lys2Δ0 trp1Δ63 ura3Δ0met15Δ0 can1::MFA1pr-HIS3 hht1-hhf1::NatMX4 hht2-hhf2::[HHTS-HHFS]*-ura3Δ H3WT NHP10-6XHA::HphNT1*	This study
YNL423	*MATa his3Δ200 leu2Δ0 lys2Δ0 trp1Δ63 ura3Δ0met15Δ0 can1::MFA1pr-HIS3 hht1-hhf1::NatMX4 hht2-hhf2::[HHTS-HHFS]*-ura3Δ H3K37A NHP10-6XHA::HphNT1*	This study

YNL439	*MATa his3Δ200 leu2Δ0 lys2Δ0 trp1Δ63 ura3Δ0met15Δ0 can1::MFA1pr-HIS3 hht1-hhf1::NatMX4 hht2-hhf2::[HHTS-HHFS]*-ura3Δ H3K37A HMO1-6XHA::HphNT1*	This study
YNL442	*MATa his3Δ200 leu2Δ0 lys2Δ0 trp1Δ63 ura3Δ0met15Δ0 can1::MFA1pr-HIS3 hht1-hhf1::NatMX4 hht2-hhf2::[HHTS-HHFS]*-ura3Δ ρ–*	This study
YNL445	*MATa his3Δ200 leu2Δ0 lys2Δ0 trp1Δ63 ura3Δ0met15Δ0 can1::MFA1pr-HIS3 hht1-hhf1::NatMX4 hht2-hhf2::[HHTS-HHFS]*-ura3Δ ρ–*	This study
Plasmid	***Description***	***Reference/Source***
pRS415	*LEU2 CEN/ARS*	(1)
pRS416	*CEN/ARS URA3*	(1)
pRS425	*LEU2* 2μ	(1)
pPL155	*HA3-TOR1* A1957V	(2)
pPHY1299	pRS425; *PDE2*	(3)
pHMO1	*GAL1-HMO1-6XHis/3XHA/Protein A URA3*	Open Biosystems
pNHP6A	*GAL1-NHP6A-6XHis/3XHA/Protein A URA3*	Open Biosystems
pNHP10	*GAL1-NHP10-6XHis/3XHA/Protein A URA3*	Open Biosystems
pABF2	*GAL1-ABF2-6XHis/3XHA/Protein A URA3*	Open Biosystems
pIXR1	*GAL1-IXR1-6XHis/3XHA/Protein A URA3*	Open Biosystems

References

1. Brachmann, C. B., A. Davies, G. J. Cost, E. Caputo, J. Li, P. Hieter, and J. D. Boeke. 1998. Designer deletion strains derived from Saccharomyces cerevisiae S288C: a useful set of strains and plasmids for PCR-mediated gene disruption and other applications. *Yeast* 14:115-132.
2. Reinke, A., J. C. Chen, S. Aronova, and T. Powers. 2006. Caffeine targets TOR complex I and provides evidence for a regulatory link between the FRB and kinase domains of Tor1p. *J Biol Chem* 281:31616-31626.
3. Howard, S. C., A. Hester, and P. K. Herman. 2003. The Ras/PKA signaling pathway may control RNA polymerase II elongation via the Spt4p/Spt5p complex in Saccharomyces cerevisiae. *Genetics* 165:1059-1070.
